# Evaluating the effectiveness of waste glass powder for the compressive strength improvement of cement mortar using experimental and machine learning methods

**DOI:** 10.1016/j.heliyon.2023.e16288

**Published:** 2023-05-13

**Authors:** Kaffayatullah Khan, Waqas Ahmad, Muhammad Nasir Amin, Muhammad Isfar Rafiq, Abdullah Mohammad Abu Arab, Inas Abdulalim Alabdullah, Hisham Alabduljabbar, Abdullah Mohamed

**Affiliations:** a Department of Civil and Environmental Engineering, College of Engineering, King Faisal University, Al-Ahsa, 31982, Saudi Arabia; b Department of Civil Engineering, COMSATS University Islamabad, Abbottabad, 22060, Pakistan; c Department of Chemistry, College of Science, King Faisal University, Al-Ahsa, 31982, Saudi Arabia; d Department of Civil Engineering, College of Engineering in Al-Kharj, Prince Sattam Bin Abdulaziz University, Al-Kharj, 11942, Saudi Arabia; e Research Centre, Future University in Egypt, New Cairo, 11835, Egypt

**Keywords:** Cement mortar, Compressive strength, Waste glass powder, AdaBoost, Decision tree

## Abstract

This study utilized both experimental testing and machine learning (ML) strategies to assess the effectiveness of waste glass powder (WGP) on the compressive strength (CS) of cement mortar. The cement-to-sand ratio was kept 1:1 with a water-to-cement ratio of 0.25. The superplasticizer content was 4% by cement mass, and the proportion of silica fume was 15%, 20%, and 25% by cement mass in three different mixes. WGP was added to cement mortar at replacement contents from 0 to 15% for sand and cement with a 2.5% increment. Initially, using an experimental method, the CS of WGP-based cement mortar at the age of 28 days was calculated. The obtained data were then used to forecast the CS using ML techniques. For CS estimation, two ML approaches, namely decision tree and AdaBoost, were applied. The ML model's performance was assessed by calculating the coefficient of determination (R^2^), performing statistical tests and k-fold validation, and assessing the variance between the experimental and model outcomes. The use of WGP enhanced the CS of cement mortar, as noted from the experimental results. Maximum CS was attained by substituting 10% WGP for cement and 15% WGP for sand. The findings of the modeling techniques demonstrated that the decision tree had a reasonable level of accuracy, while the AdaBoost predicted the CS of WGP-based cement mortar with a higher level of accuracy. Utilizing ML approaches will benefit the construction industry by providing efficient and economic approaches for assessing the properties of materials.

## Graphical abstract

**Figure:**
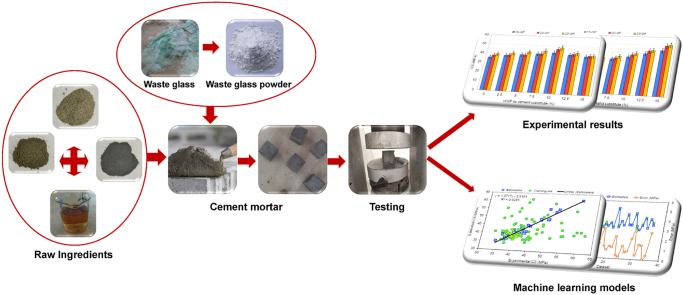

## 1 Introduction

Several processes, such as manufacturing, steel and iron metallurgy, electricity generation, mining, agricultural production, and the creation of electronic appliances, make immense volumes of solid waste. Several harmful solid wastes are infectious, flammable, chemically reactive, incendiary, and corrosive; their disposal in landfill areas has resulted in extensive economic failures [1,2]. Consequently, it is appropriate to recycle solid waste in construction materials [[3], [4], [5]]. Cementitious composites are extensively used in the building industry [[6], [7], [8], [9], [10], [11]]. Numerous approaches have been utilized by academics to enhance the efficiency of cementitious composites [[11], [12], [13], [14], [15], [16]]. Also, to produce eco-friendly materials, waste materials might be incorporated as replacements for aggregate [[17], [18], [19]], cement [20,21], and reinforcing fibers [[22], [23], [24], [25], [26], [27], [28]]. The use of recycled aggregate concrete and fiber-reinforced recycled aggregate concrete is gaining popularity [29,30]. Due to the lower use of cement and aggregates, natural resources and CO_2_ emissions may be conserved [[31], [32], [33]]. It has been reported that utilizing waste materials in cementitious composites improved their mechanical characteristics [34]. Globally, a large quantity of waste glass (WG) is produced, with a considerable proportion of WG disposed of in landfill areas [35]. The rising population is creating more municipal waste, and landfill area is becoming increasingly scarce, mainly in big municipalities [36]. In comparison with other wastes like wood and plastic, WG is chemically durable. WG is non-biodegradable even if buried for a longer time [37]. Additionally, some glass types, e.g., cathode ray tube (CRT), comprise poisonous elements like mercury, cadmium, beryllium, and lead, contaminating underground water and soil [38]. China makes around 43 million tons of CRT glass each year [39], presenting a significant ecological risk and compromising human health.

To manufacture glass, silica needs to be melted at an elevated temperature and needs considerable energy [40]. The temperature is retained at 1500 °C for 72 h for plate glass and 24 h for container glass [41]. One kg of plate glass uses roughly 17 J of fossil fuel energy and releases around 600 g of CO_2_ [42]. The yearly energy use for glass production in Europe exceeds 350 PJ, which is around 20% of the overall industrial energy usage [42]. Thus, the appropriate recycling of WG is attracting major worldwide interest. Reprocessing WG to produce glass goods is a prevalent approach to WG reuse. Nevertheless, reprocessing is complex. In order to create glass containers and plates, WG must be separated, cleaned, and melted [43]. The production of building materials from WG is another form of WG recycling. WG may be crushed and used to partially replace sand and cement in cementitious composites [44,45]. Utilizing WG in cementitious composites has several benefits. First, WG does not require melting, which decreases energy use. Second, WG is easily managed, as there is no necessity to clean and sift WG. Thirdly, the extensive use of cementitious composites in building materials will increase the need for WG. Cementitious composites can encapsulate and solidify the toxic components of glass. Previous exploration has proved that WG recycling in cementitious composites is a better technique [44]. Consequently, the utilization of WG as a replacement for sand in building materials will conserve natural raw materials and simplify waste managing. In an identical manner, WG utilization as a cement substitute will help decrease cement demand and, accordingly, CO_2_ emissions, it will also reduce cement demand [46,47].

Numerous research is conducted to evaluate the properties of cementitious composites; nevertheless, compressive strength (CS), particularly 28-day CS, is regarded as important [31]. The CS of cementitious composites gives vital knowledge of its many characteristics [48,49]. The CS of cementitious composites is linked to its various other characteristics [50]. At present, analytical approaches for the material characteristics are being employed to reduce unnecessary resource consumption and tests. Because of the non-linear nature of cementitious composites in compression [51,52], regression algorithms built in this approach may not adequately characterize the inherent material behavior. Additionally, regression techniques might overstate the implication of some features [53]. Artificial intelligence (AI)-based techniques, like supervised machine learning (ML), are among the highly advanced prediction approaches for the stated purpose [[54], [55], [56], [57], [58]]. ML strategies simulate outcomes on the basis of input features, and testing confirms the resultant models [[59], [60], [61]]. Using ML methods to forecast the characteristics of cementitious composites and bituminous mixtures is gaining popularity [[62], [63], [64]]. The bulk of previous ML research concentrated on predicting the CS of normal cementitious composites [[65], [66], [67], [68]], whereas just a few studies predicted the features of cementitious composites with WG.

This study evaluated the effect of incorporating waste glass powder (WGP) on the CS of cement mortar using experimental and theoretical methods. Cement mortar specimens were cast with varying amounts of WGP as a sand and cement substitute (0–15%). The CS of WGP-modified cement mortar at 28 days was evaluated using experimental methods. After the experimental tests, the generated dataset was used to build ML-based estimation models of the CS of mortar. To accomplish the study's objectives, the decision tree (DT) and AdaBoost regressor (AR) techniques were employed. AR is an ensemble ML method, whereas DT is a single ML method. The ML model's accuracy was evaluated and compared via the coefficient of determination (R^2^), statistical tests, the k-fold validation, and the difference in estimated results. This study is novel in that it assessed the CS of WGP-modified cement mortar via both experimental testing and ML models. Material gathering, specimen casting, curing, and performing experiments demand considerable time, expense, and effort in experimental studies. The construction industry will benefit from addressing these obstacles with cutting-edge techniques like ML. Therefore, the purpose of this study was to increase knowledge regarding the use of ML methods to predict material characteristics. ML techniques require a data sample, which can be generated through an experimental approach or from literature. The data acquired may therefore be used to train ML methods and estimate material attributes. This research utilized six input factors to estimate the CS WGP-modified cement mortar WGP and assess the efficacy of each ML model.

## 2 Methods

### 2.1 Experimental setup

**Fig. 1:**
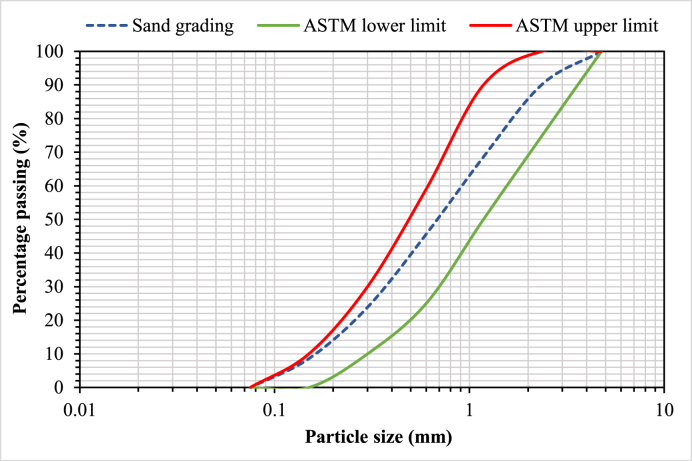
Particle size distribution of sand used in the present study.

**Fig. 2:**
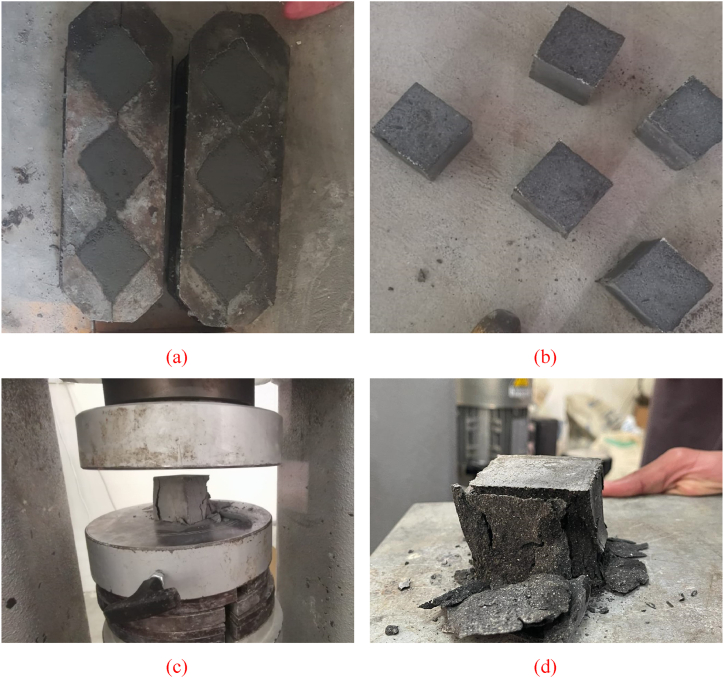
Experimental program images at different stages: (a) Specimens in molds; (b) Demolded specimens; (c) Compression testing; (e) Tested specimen.

Locally available Portland cement and sand were collected, whilst silica fume and superplasticizer were acquired from Pakistan's PAGEL Construction Chemicals. The particle size distribution of sand used is provided in Fig. 1. Float WG was collected from local construction rubble, washed, and crushed into a powder before passing through sieve #200. As shown in Table 1, three separate control mix designs were selected for cement mortar. Silica fume was added to the control mixes to study only the effect of WGP on the CS of cement mortar. In addition, WGP was added to all mix designs in proportions of 2.5, 5, 7.5, 10, 12.5, and 15% as a cement and sand replacement in all mixes. The ingredients were mixed via the mechanical mixer. Water and superplasticizer were put together. Cement, silica fume, sand, and WGP were added to the mixer pan, followed by the addition of half of the water and 2 min of mixing. The remaining half of the water was added in two additions while the mixer continued to rotate for 2 min. The overall duration of mixing was 4 min. To test the CS, 50 mm cube specimens were cast. For each formulation, three specimens were cast, and an overall 117 samples were cast and evaluated. After being cast, specimens were stored at room temperature for 24 h before being demolded and placed in water to cure. All specimens were cured in water for 28 days. The CS test was conducted in accordance with ASTM C109/C109M − 20 [69] utilizing a 1000 kN load-controlled compression testing equipment. Fig. 2 depicts images of specimens and test setup.

**Table 1:** Raw ingredients used in different mixes.

Constituent (kg/m^3^)	Type of control mix		
	15-SF	20-SF	25-SF
Cement	810	760	720
Water	203	191	180
Sand	810	760	720
Superplasticizer	40.5	38	36
Silica fume	122	153	180

### 2.2 Modeling

**Fig. 3:**
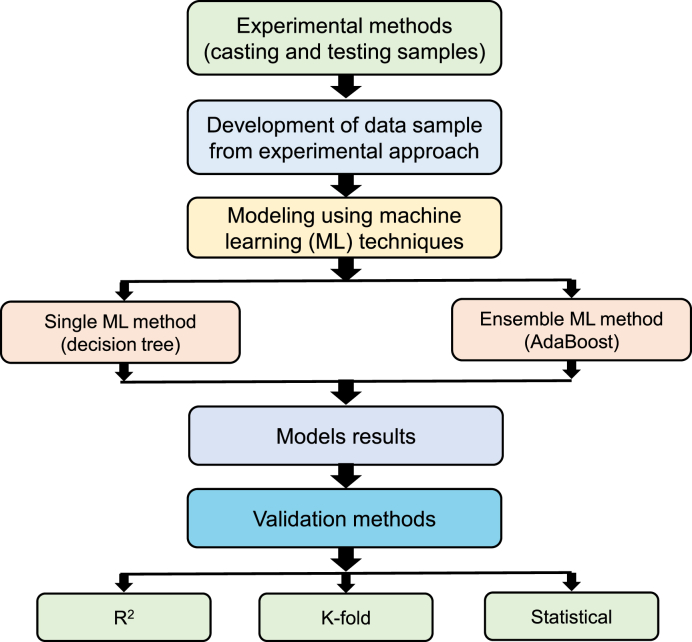
Flow diagram indicating the sequence of research methods.

To get the desired findings, ML approaches require a broad array of input variables [70]. Using experimental dataset, the CS of cement mortar was determined. The algorithms used cement, water, sand, silica fume, superplasticizer and WGP as inputs and CS as an output. Table 2 displays the statistical parameters for all inputs and outputs used for running algorithms. Single and ensemble ML algorithms with Python code and Spyder (version 5.1.5) from the Anaconda Navigator software were used to attain the objectives of the study. DT was employed as an individual ML algorithm, while AR was employed as an ensemble ML technique. Normally, these ML procedures are applied to assess required findings using input features. These ML methods might be utilized to assess a material's performance [71,72]. The percentage of the dataset used for modeling was 70% for training and 30% for testing. R^2^ is the proportion of the dependent variable's variance that is predicted by the statistical model. The R^2^ of the resultant model shows its accuracy. The R^2^ shows the level of deviation; a value near 0 denotes a larger disparity, while a value near 1 indicates that the estimated results and actual data are nearly perfectly matched [73]. K-fold, statistical, and error assessments, including root mean square error (RMSE), mean absolute error (MAE), and mean absolute percentage error (MAPE) were carried out on the employed ML techniques. Fig. 3 illustrates a flowchart of research methods.

**Table 2:** Statistical factors of input and output variables.

Parameter	Input variables						Output variable
	Cement (kg/m^3^)	Water (kg/m^3^)	Sand (kg/m^3^)	Superplasticizer (kg/m^3^)	Silica fume (kg/m^3^)	WGP (kg/m^3^)	CS (MPa)
Minimum	612	180	612	36	122	0	34.24
Maximum	810	203	810	40.5	180	121.5	62.46
Mean	732.51	191.33	735.62	38.17	151.67	61.65	43.48
Standard Deviation	53.62	9.43	54.51	1.85	23.8	36.34	6.09
Standard Error	4.96	0.87	5.04	0.17	2.2	3.36	0.56
Mode	810	203	810	40.5	122	0	40.2
Median	722	191	729	38	153	60.75	41.4

### 2.3 Model's validation

**Fig. 4:**
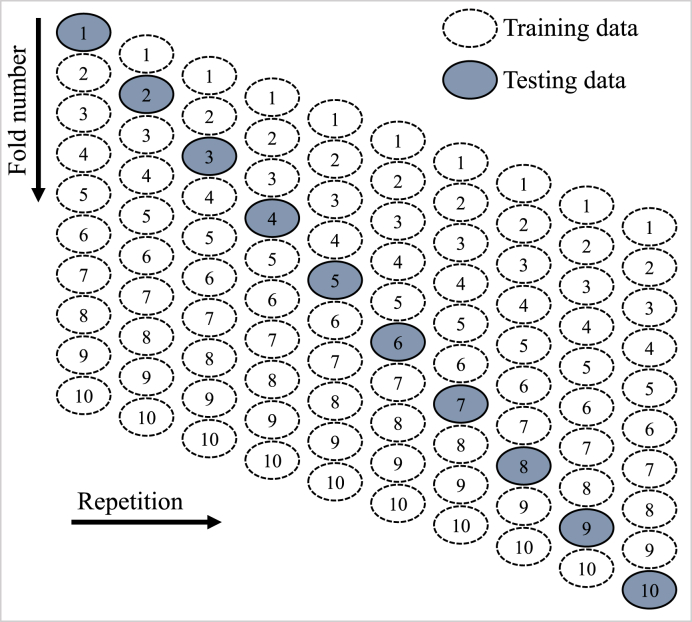
K-fold approach running process.

The built ML models were assessed using statistical and k-fold methods. Generally, the k-fold validation is performed to evaluate the efficacy of a model by randomly dividing the data sample into 10 groups [74]. Fig. 4 shows that nine classes were utilized to train ML techniques, with just one utilized for testing. When the errors are less, and R^2^ is greater, the ML model is more accurate. In addition, 10 repeats of the technique are necessary for the desired impact. This effort is a major factor in the very accurate prediction of the model. Additionally, errors assessment, like MAE, RMSE, and MEPE, was used to statistically test the precision of each ML model. Eqs. (1), (2), (3), derived from the literature [75,76], were utilized to statistically assess the estimation accuracy of the ML models.

(1) $$MAE=\frac{1}{n}\sum_{i=1}^{n}\left|P_{i}-Ei\right|$$

(2) $$RMSE=\sqrt{\sum \frac{{\left(P_{i}-E_{i}\right)}^{2}}{n}}$$

(3) $$MAPE=\frac{100\%}{n}\sum_{i=1}^{n}\frac{\left|P_{i}-Ei\right|}{E_{i}}$$

where n = number of data samples, $P_{i}$ = predicted CS, and $E_{i}$ = experimental CS.

## 3 Results and analysis

### 3.1 Experimental CS

**Fig. 5:**
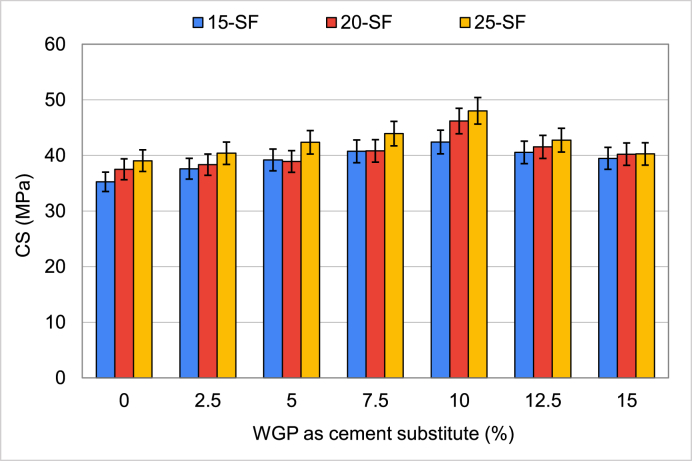
Results of CS when WGP was used as a cement substitute.

The CS of specimens that contains WGS as a cement replacement is displayed in Fig. 5. The improvement of CS was foreseen after the inclusion of WGP. Increasing the WGP concentration up to 10% of cement in all mixes enhanced the CS, but beyond that point, the CS began to decrease. Compared to the control sample (0% WGP), the CS of the specimens containing 12.5 and 15% WGP as cement substitutes were likewise higher than the reference specimen. The percentage increase in CS of cement mortar in 15-SF mixes was 6.7% for 2.5% WGP, 11.2% for 5% WGP, 15.6% for 10% WGP, 20.3% for 15% WGP, and 11.9% for 15% WGP higher than the reference sample (0% WGP). Although similar outcomes were seen with different mixtures (20-SF and 25-SF), the highest CS was attained with 10% WGP ratio as a cement substitute, which was about 23% greater than the control sample. The filler effect and pozzolanic characteristics of WGP are two probable reasons [35]. Because of the filler effect, the porosity is reduced, and the resultant matrix is dense and compact. When Ca(OH)_2_ in the cement mix combines with SiO_2_ in the glass's chemical composition [77], a thick calcium-silicate-hydrate (C–S–H) gel is formed, enhancing the material's performance [78,79]. When more WGP than is needed for the pozzolanic reaction is added to the mixture, the CS drops, as seen at higher concentrations of WGP (12.5% and 15%) [35]. Thus, using WGP as a cement substitute of up to 10% is beneficial for the highest strength.

**Fig. 6:**
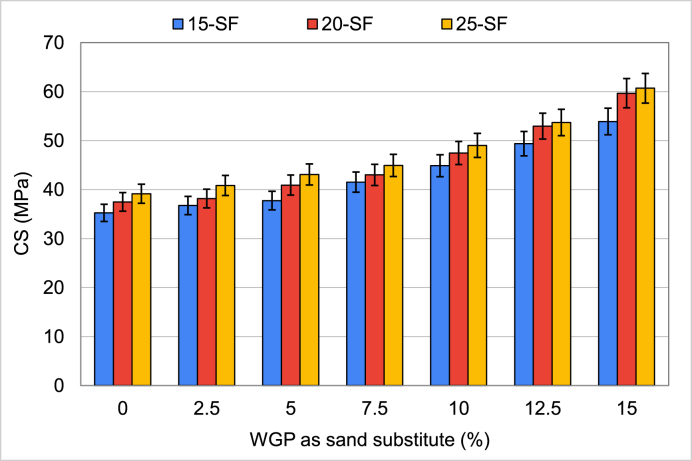
Results of CS when WGP was used as a sand substitute.

Results of specimens with WGP used as sand replacement are shown in Fig. 6. All three mixtures benefited from WGP's inclusion; the CS improved with greater amounts. When compared to the control sample, the CS of samples incorporating WGP at 2.5%, 5%, 7.5%, 10%, 12.5%, and 15% contents in the 15-SF mix increased by 4.3%, 7.1%, 17.8%, 27.3%, 40.1%, and 53%, respectively. Likewise, in 20-SF and 25-SF, the CS increased with increasing WGP content, and the highest CS was attained with 15% WGP as a sand substitute. At a 15% replacement ratio, the CS improvement was approximately 59% in 20-SF mixes and 55% in 25-SF mixes. Since WGP was finer than aggregate, this may have been the primary factor in the CS improvement [80]. The pozzolanic reaction caused by the addition of WGP to the cement produced enhanced hydration products such as C–S–H gel, which in turn increased the material's CS [35]. Hence, WGP could be incorporated up to a 15% replacement ratio as a sand substitute to attain the highest CS.

### 3.2 Prediction models

#### 3.2.1 DT model

**Fig. 7:**
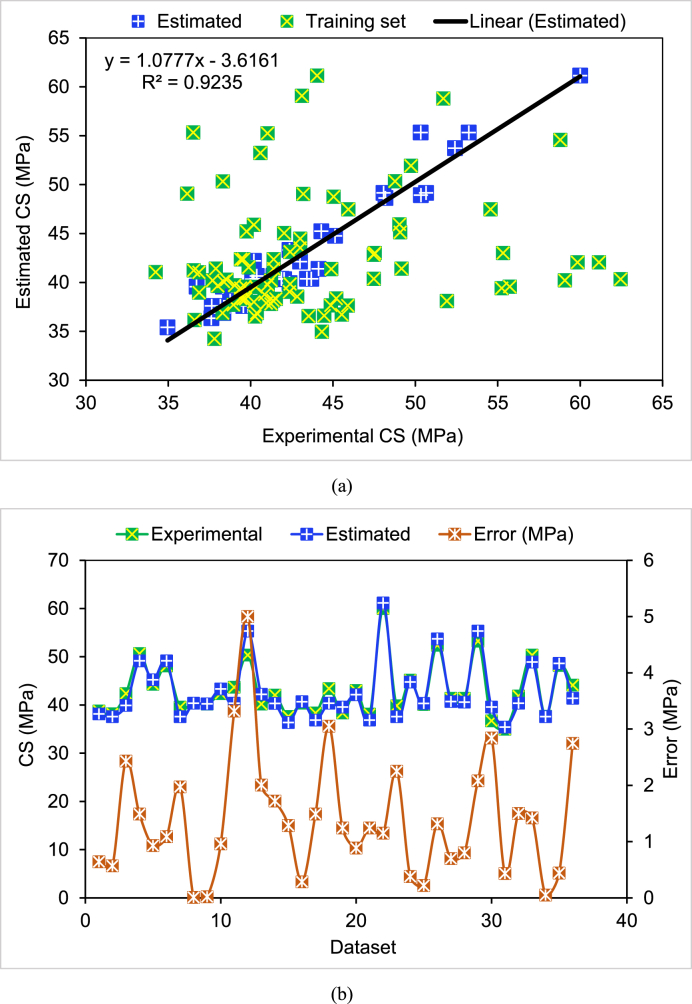
Decision tree model: (a) Link between actual and estimated CS; (b) Dispersion of experimental and estimated CS and errors.

Outcomes of the DT model to anticipate the CS of cement mortar with distinct proportions of WGP are shown in Fig. 7. Fig. 7(a) portrays the link between experimental and predicted CS. The results found by using the DT method were quite accurate, with minimal variation among the actual and predicted CS. The R^2^ of 0.92 indicated that the DT approach for predicting the CS of cement mortar containing WGP is excellent and that the experimental and anticipated findings are in good agreement. Readings of experimental, predicted, and divergent (errors) values from the DT method are shown in Fig. 7(b). The error values distribution was from 0.01 to 5.0 MPa with a mean of 1.39 MPa. It was also noted that 47.2% of the error values ranged within 1–3 MPa, while 8.3% were greater than 3 MPa, and 41.7% were less than 1 MPa. The CS of cement mortar incorporating WGP was correctly predicted using the DT approach, as shown by the distribution errors.

#### 3.2.2 AdaBoost model

**Fig. 8:**
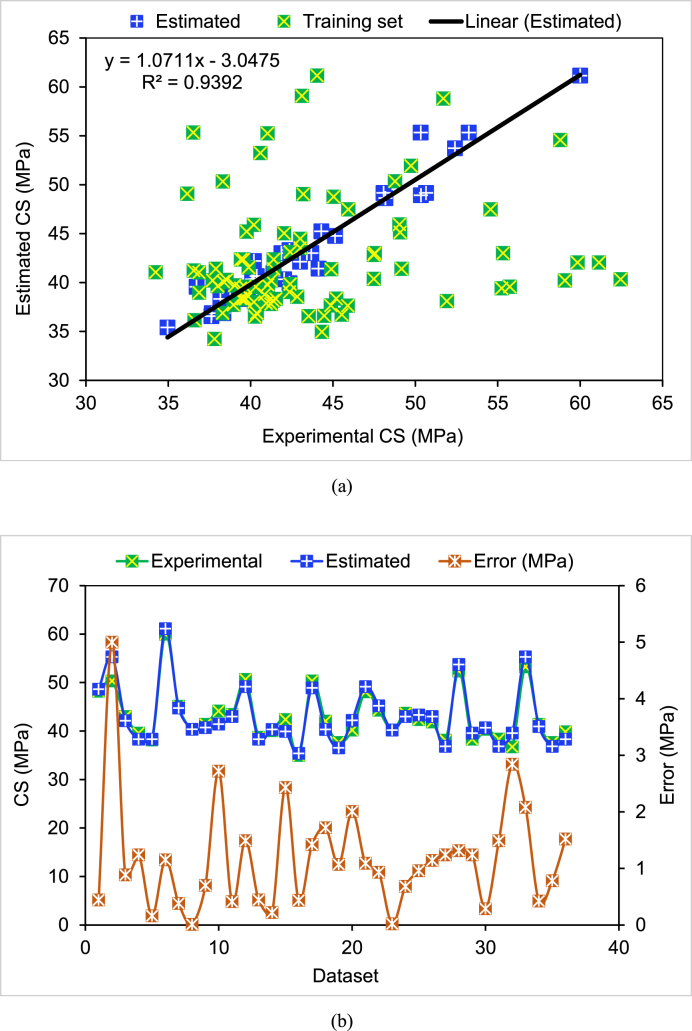
AdaBoost model: (a) Link between actual and estimated CS; (b) Dispersion of experimental and estimated CS and errors.

The results of the AR model to forecast the CS of cement mortar containing WGP are exhibited in Fig. 8. Link between real and forecasted CS is seen in Fig. 8(a). The AR method resulted in the least amount of variation between experimental and estimated CS, making it the more accurate. A greater R^2^ for the AR model indicated better accuracy. The scatter plot of experimental, estimated, and errors for the AR method is shown in Fig. 8(b). Errors ranging from 0.01 to 5.0 MPa were determined for the AR model. According to the assessment of the errors, almost 47.2% of values were found to be less than 1 MPa, nearly 50% to be between 1 and 3 MPa, and only 2.8% to be above 3 MPa. Error statistics further supported the AR model's superior precision over the DT model. Due to its use of an infinite number of DTs during training, the AR model had a higher accuracy. The same data records were also used by a different model. This procedure was repeated until enough novice learners had been formed. Furthermore, AR improved the effectiveness of DTs for binary classification.

### 3.3 Model's validation

**Fig. 9:**
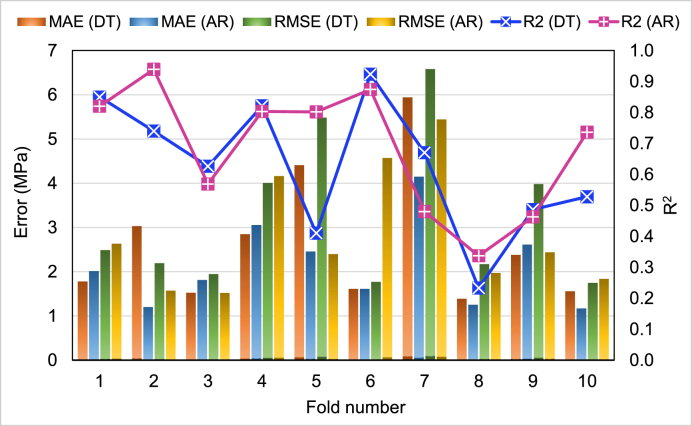
Comparison of R^2^, RMSE, and MAE for the employed ML models from the k-fold analysis.

Table 3 displays the findings of error assessments (MAE, MAPE, and RMSE). The MAE values for DT and AR were found to be 1.388 and 1.167 MPa, respectively. Similarly, MAPE was calculated to be 3.2% for DT and 2.7% for AR. In addition, the RMSE for DT was computed to be 1.747 MPa, while the RMSE for AR was found to be 1.519 MPa. Based on these evaluations, the AR model seems to be more precise than DT. Table 4 shows the resulting R^2^, MAE, and RMSE values that were used to validate models via the k-fold method. Fig. 9 was made to compare the results of both ML methods' k-fold assessment. Using the DT method, the MAE values varied from 1.39 to 5.94 MPa, with an average of 2.65 MPa. The AR approach's MAE varied from 1.17 to 4.15 MPa, with an average of 2.15 MPa. Comparatively, the RMSE for the DT and AR techniques averaged 3.24 and 2.85 MPa. On the other hand, the average R^2^ for DT and AR models were 0.63 and 0.68, respectively. The CS of cement mortar containing WGP may be estimated with the optimum precision using the AR approach with the lowest error rates and the highest R^2^ values.

**Table 3:** Findings of statistical assessment indicating MAE, MAPE, and RMSE for the employed models.

Technique	MAPE (%)	MAE (MPa)	RMSE (MPa)
Decision tree	3.20	1.388	1.747
AdaBoost regressor	2.70	1.167	1.519

**Table 4:** Readings of MAE, RMSE, and R^2^ for the ML methods from the k-fold approach.

K-fold	Decision tree			AdaBoost regressor		
	R^2^	RMSE (MPa)	MAE (MPa)	R^2^	RMSE (MPa)	MAE (MPa)
1	0.85	2.49	1.78	0.82	2.63	2.01
2	0.74	2.19	3.03	0.94	1.57	1.2
3	0.63	1.95	1.52	0.57	1.52	1.82
4	0.82	4	2.85	0.8	4.16	3.05
5	0.41	5.48	4.41	0.8	2.4	2.46
6	0.92	1.77	1.61	0.87	4.57	1.61
7	0.67	6.58	5.94	0.48	5.44	4.15
8	0.23	2.17	1.39	0.34	1.97	1.25
9	0.49	3.98	2.38	0.46	2.44	2.61
10	0.53	1.75	1.56	0.74	1.84	1.17

## 4 Discussions

This research examined the influence of utilizing WGP on the CS of cement mortar by both experimental and modeling methods. Worldwide, a substantial amount of WG is produced, and most of it ends up in landfills, where it puts dangers to both human and environmental health [35]. Furthermore, cementitious composites are the most widely used building materials [[81], [82], [83]], but their increased consumption results in the loss of natural resources and the release of CO_2_. To reduce environmental impact, WG might be used to partially substitute cement and sand in cementitious composites. In this way, the utilization of WG in cementitious composites will lessen its detrimental impacts on the environment by preventing waste, saving resources, and producing less CO_2_. This research aimed to fill the knowledge gap by combining experimental and ML-based modeling techniques to learn more about WGP's role in cementitious composites. Cement mortar samples were cast with varying amounts of WGP (0–15%) in place of cement and sand. The experimental tests showed that the CS of cement mortar was enhanced by the addition of WGP. At 10% WGP content as cement replacement, the CS was optimum, resulting in a 23% increase over the reference sample. A relevant study by Anwar [84] also found enhancement in the CS of cementitious composites when WGP was used in lower concentrations. The optimum proportion of WGP as a cement substitute was noted to be 10%, resulting in around a 16.6% rise in CS that the reference sample. Similarly, in another study by Aliabdo et al. [45], the optimum WGP content as a cement substitute in cementitious composites was recorded at 10%, causing around a 4.8% increase in CS compared to the reference sample in 45 MPa concrete. Similar findings were also reported by Kamali and Ghahremaninezhad [85]. Hence, this research's findings were found to be comparable with several past studies. The filler effect and WGP's pozzolanic property were two possible explanations. The matrix was compact and dense due to the filler effect, which decreased porosity. More SiO_2_ in the glass combined with Ca(OH)_2_ in the cement hydration product to produce dense C–S–H gel, which improved the material's characteristics [78,79]. When WGP was used at concentrations of 12.5 and 15% as a cement substitute, the CS decreased because extra WGP was being added than required for the pozzolanic process [35]. Cement replacement of up to 10% by WGP is thus advised for maximum strength. When WGP was incorporated as a sand substitute in cement mortar, the CS was up to 59% higher than the reference sample at a 15% replacement level. As WGP had finer particles than sand, the improvement in particle packing may explain the increase in CS [80]. WGP's pozzolanic interaction with cement increased the development of beneficial hydration products such as C–S–H gel, which in turn increased the material's CS [35]. Therefore, to get the highest possible strength, WGP might be utilized as a sand alternative at a 15% ratio. However, further investigations are required to assess WGP's impact at higher proportions.

The ML models were executed after the experimental data was organized. The CS of WGP-containing cement mortar was projected using two ML strategies: single (DT) and ensemble (AR). Both models' predictivity was analyzed to see which one was superior. When compared to the DT's R^2^ of 0.92, the AR model's R^2^ of 0.94 indicated more precision. Lower readings for the MAE, MAPE, and RMSE also favored the AR model, further confirming its superior accuracy. However, the DT model also produced findings that were reasonable and consistent with the actual results. Studies from the past have also revealed how the AR model is more accurate than the DT at forecasting the strength features of various types of cementitious composites [86,87]. Wang et al. [86] used DT, AR and random forest methods to develop prediction models for the CS of geopolymer composites. After the comparison of DT and AR models, it was concluded that the AR model with an R^2^ of 0.90 was more accurate than the DT with an R^2^ of 0.83. Likewise, Shang et al. [87] developed DT and AR prediction models for the CS of recycled aggregate concrete. The results showed the AR model's superior accuracy, with an R^2^ of 0.95, compared to the DT model, with an R^2^ of 0.93. However, recognizing and recommending the best ML method for estimating results in different research fields is difficult as the effectiveness of an ML strategy is greatly reliant on the number of inputs and database size utilized to run algorithms [75]. By building sub-models that are trained on the database and revised to improve R^2^, ensemble ML techniques repeatedly utilize the weak learner compared to the single ML algorithms. Therefore, the ensemble ML techniques produce more accurate results. Exploration of this nature will help the building sector since it will spur the development of quick and low-cost strategies for assessing material characteristics.

## 5 Conclusions

This research set out to evaluate the CS of cement mortar containing varying amounts of waste glass powder (WGP) vis experimental and ML modeling techniques. The CS of specimens was determined via experiments, and the resulting data was fed into ML algorithms. The CS was predicted using decision tree (DT) and AdaBoost regressor (AR) machine learning (ML) techniques. The main findings of the study are as follows.

i. The CS of cement mortar was found to be improved with the inclusion of WGP, as shown by experimental tests. At 10% WGP content as a cement substitute, the CS was up to 23% higher than the reference specimen, representing the greatest CS achieved. Moreover, when WGP was used to replace sand, the greatest CS was achieved when 15% WGP was used, and CS was up to 59% higher than the reference sample. Possible causes for the improvement in CS include the pozzolanic activity of the glass and the filler effect, which allowed for better particle packing and reduced porosity.
ii. The outcomes of the ML models revealed that the DT model was accurate to a decent degree (R^2^ = 0.92), while the AR model was more accurate (R^2^ = 0.94) in predicting the CS of WGP-based cement mortar.
iii. For both DT and AR models, it was found that the average error in CS estimate was 1.39 MPa and 1.18 MPa, respectively. These error assessments demonstrated that both the DT and AR models were accurate to a satisfactory degree.
iv. The performance of the ML model was validated by statistical and k-fold evaluations. An accurate ML model will have lower error rates and a higher R^2^ value. A 3.2% MAPE for the DT model and a 2.7% MAPE for the AR model was found, which further confirmed the higher accuracy of the AR model.
v. Incorporating waste glass in building materials would support sustainable development by lowering environmental concerns associated with the disposal of waste glass, preventing natural resources, and decreasing CO_2_ discharges to the atmosphere.
vi. The applications of cutting-edge techniques to the construction sector, like ML modeling pave the way for more efficient and cost-effective methods of determining material characteristics.

This study was focused on exploring the compressive strength of cement mortar incorporating WGP as partial cement and sand substitute. However, for the practical applications of WGP-modified cement mortar in the construction sector, other crucial properties like durability, porosity, abrasion, frost, and carbonization resistance need to be investigated in future studies.

## Funding

This work was supported by the Deanship of Scientific Research, Vice Presidency for Graduate Studies and Scientific Research, King Faisal University, Saudi Arabia [Project No. GRANT3429].

## Author contribution statement

Kaffayatullah Khan; Inas Abdulalim Alabdullah: Conceived and designed the experiments; Wrote the paper.

Waqas Ahmad: Conceived and designed the experiments; Analyzed and interpreted the data; Contributed reagents, materials, analysis tools or data; Wrote the paper.

Muhammad Nasir Amin; Abdullah Mohamed: Analyzed and interpreted the data; Wrote the paper.

Muhammad Isfar Rafiq: Performed the experiments.

Abdullah Mohammad Abu Arab:Contributed reagents, materials, analysis tools or data.

Hisham Alabduljabbar: Contributed reagents, materials, analysis tools or data; Wrote the paper.

## Data availability statement

No data was used for the research described in the article.

## Declaration of competing interest

The authors declare that they have no known competing financial interests or personal relationships that could have appeared to influence the work reported in this paper.
